# Supplementary material for: RFWD3 and translesion DNA polymerases contribute to PCNA modification–dependent DNA damage tolerance
Source: Life Sci Alliance. 2022 Jul 29;5(12):e202201584. doi: 10.26508/lsa.202201584 (PMC9348633; doi:10.26508/lsa.202201584)
Supplement: Supplementary file 1 [file LSA-2022-01584_SdataFS1_FS3_FS4_FS5.pdf]

Fig. S1A

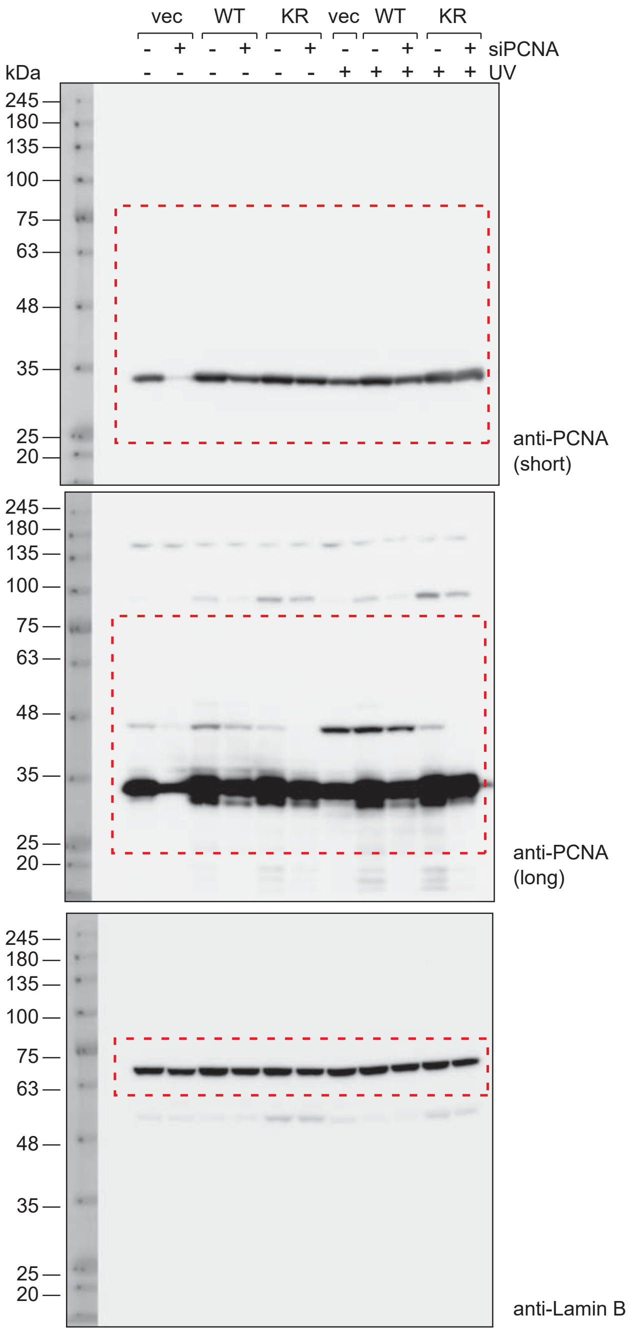
vec WT KR vec WT KR
siPCNA
UV
kDa
245
180
135
100
75
63
48
35
25
20
anti-PCNA (short)
anti-PCNA (long)
anti-Lamin B

Fig. S1H

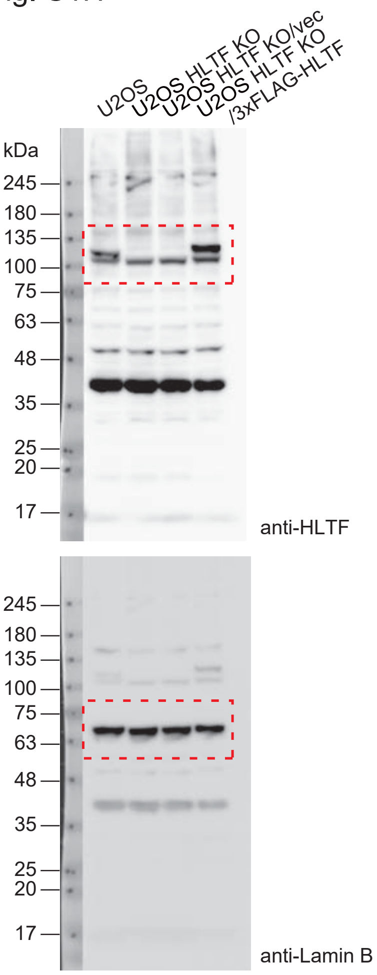

Fig. S1I

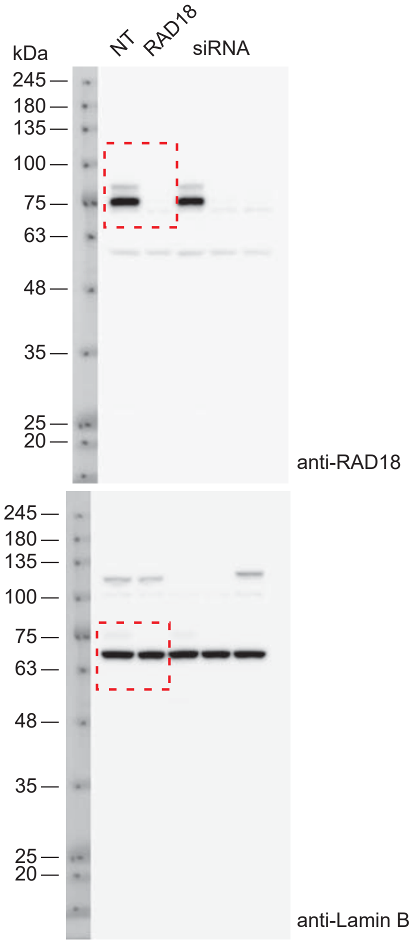

Fig. S1K

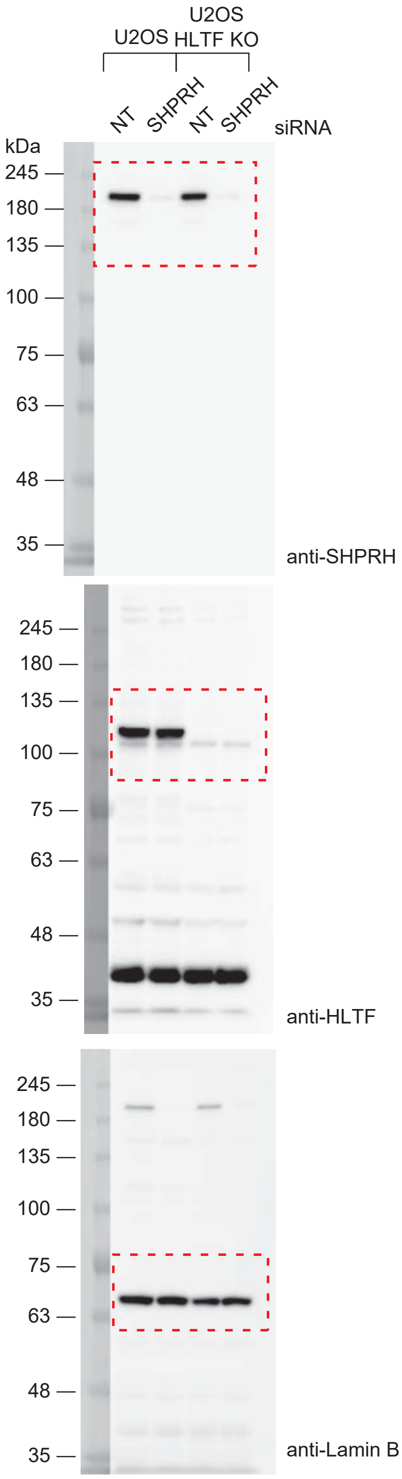

Fig. S1M

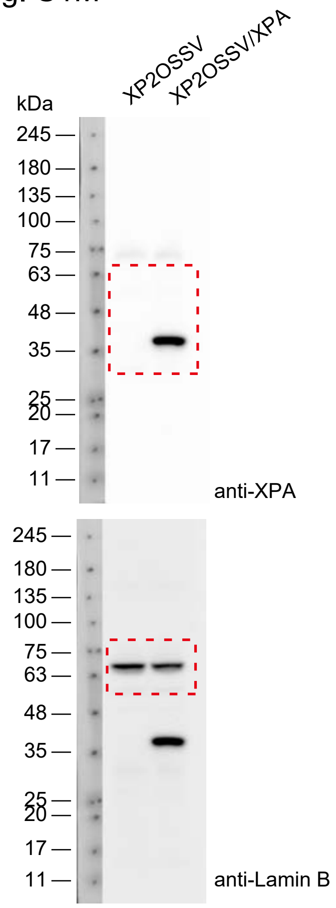

Fig. S1O

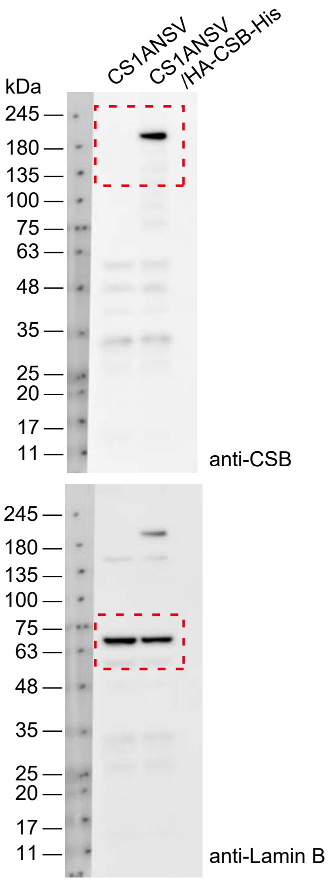

Fig. S1R

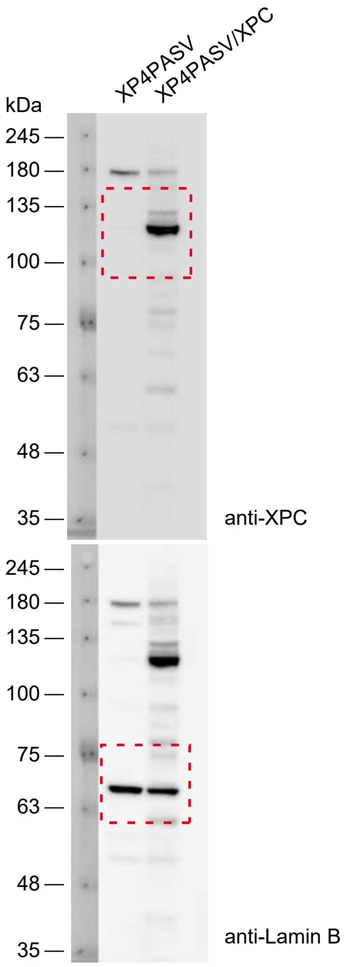

Fig. S3A

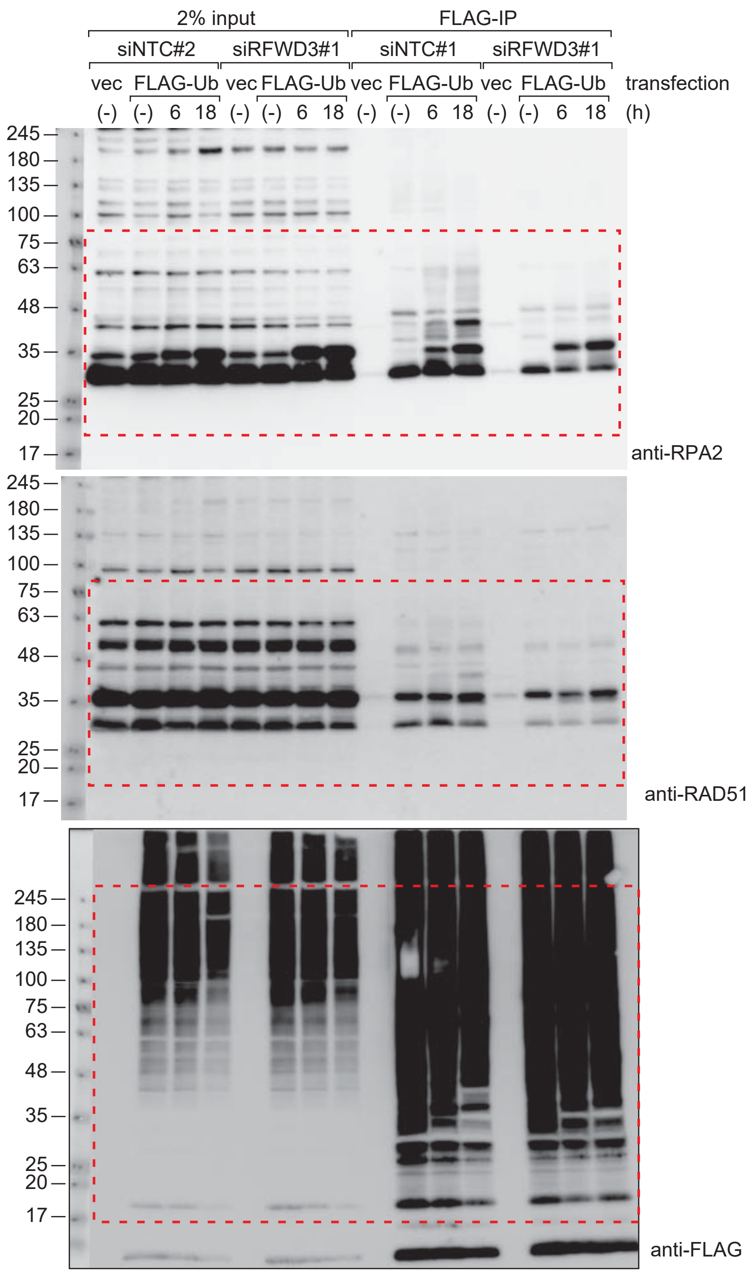

Fig. S3B

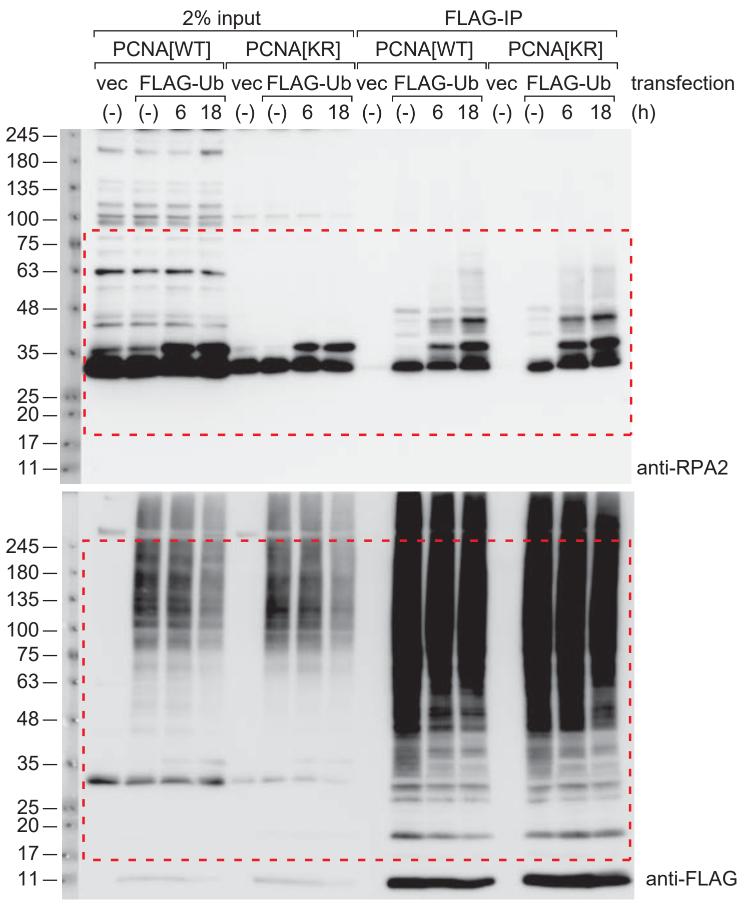

Fig. S3D

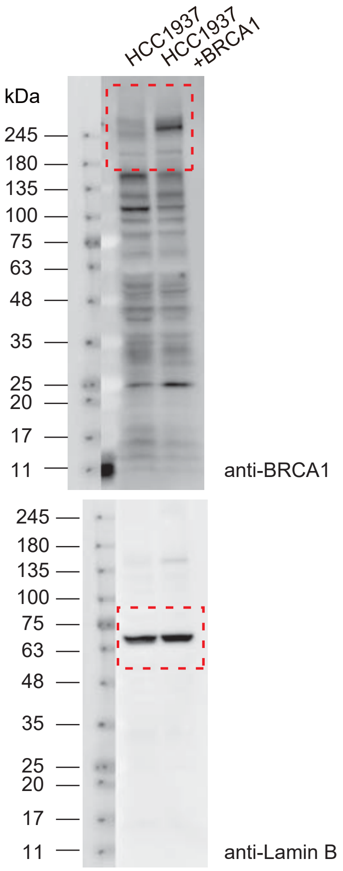

Fig. S3F

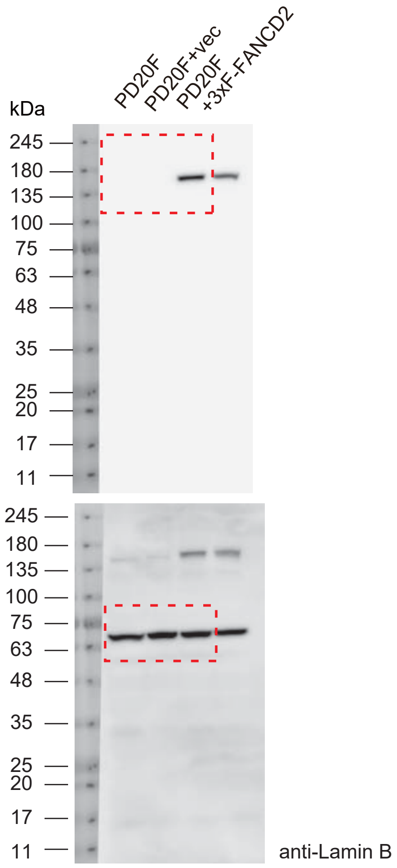

Fig. S3L

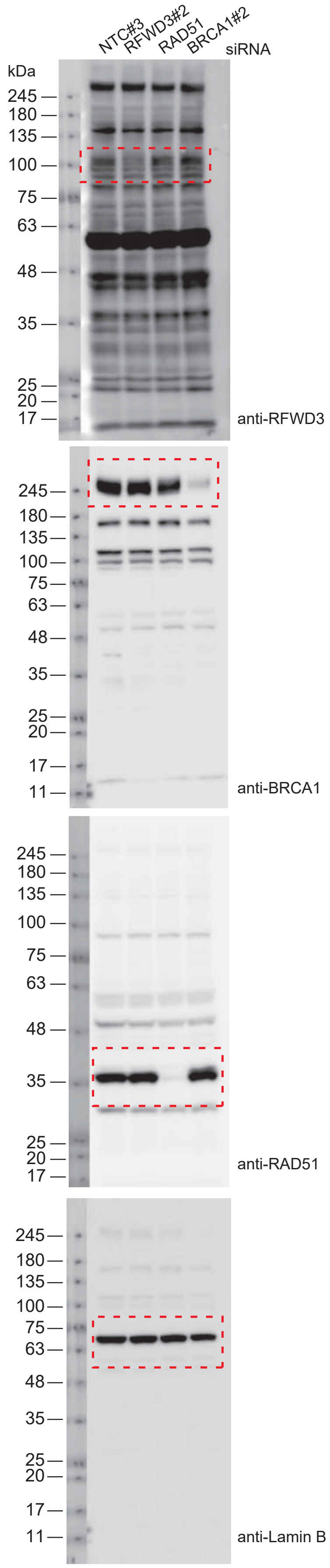

Fig. S3J

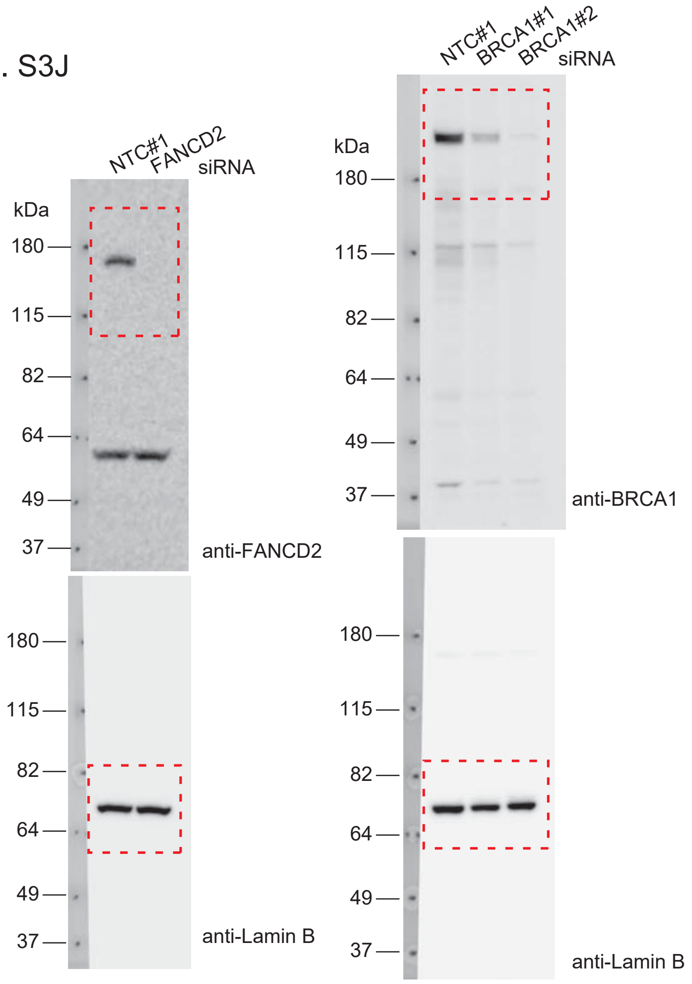

Fig. S4A

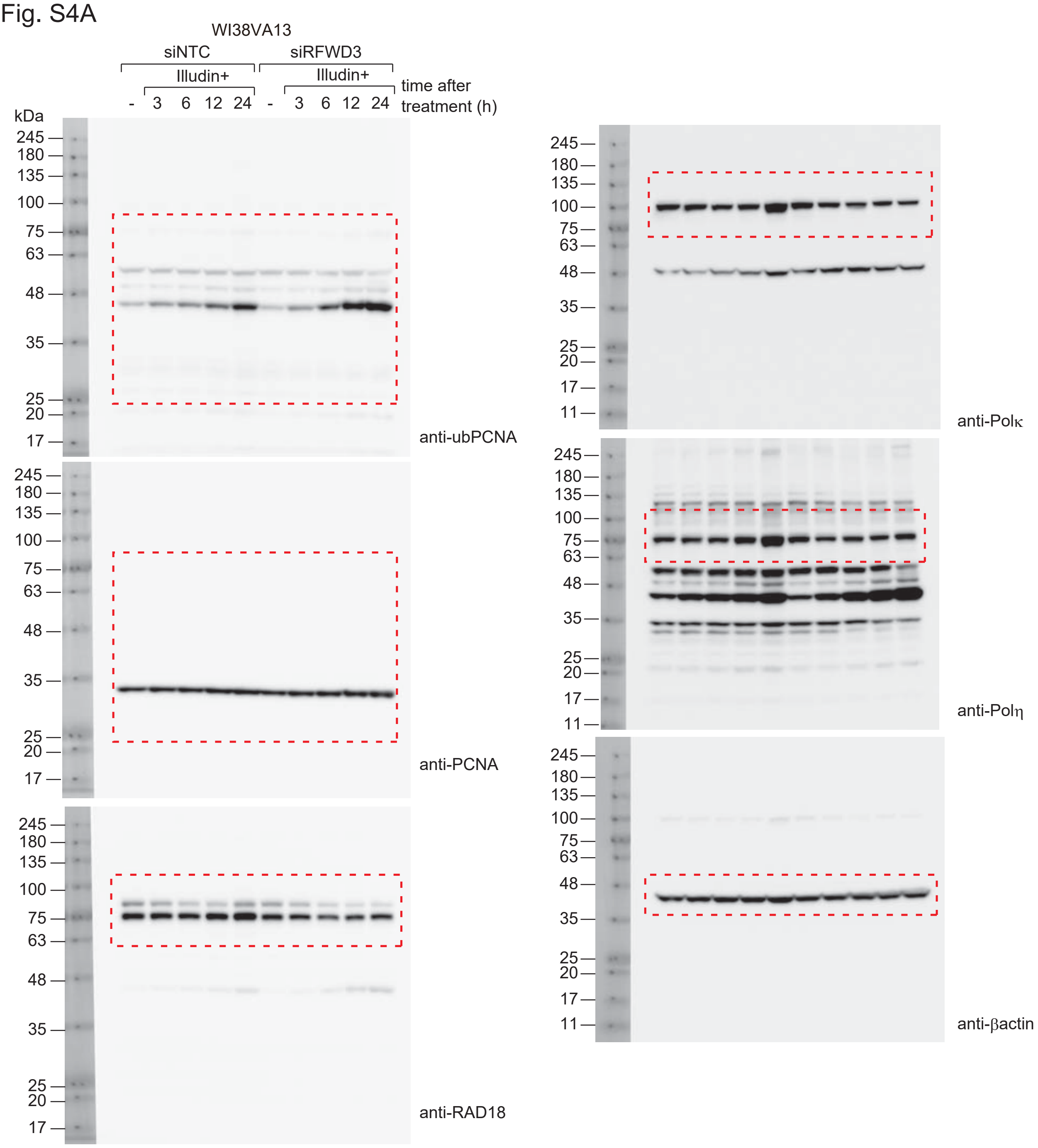
WI38VA13
siNTC
siRFWD3
Illudin+
Illudin+
- 3 6 12 24 - 3 6 12 24
time after treatment (h)
kDa
245
180
135
100
75
63
48
35
25
20
17
11
anti-ubPCNA
anti-PCNA
anti-RAD18
anti-Polκ
anti-Polη
anti-βactin

Fig. S4C

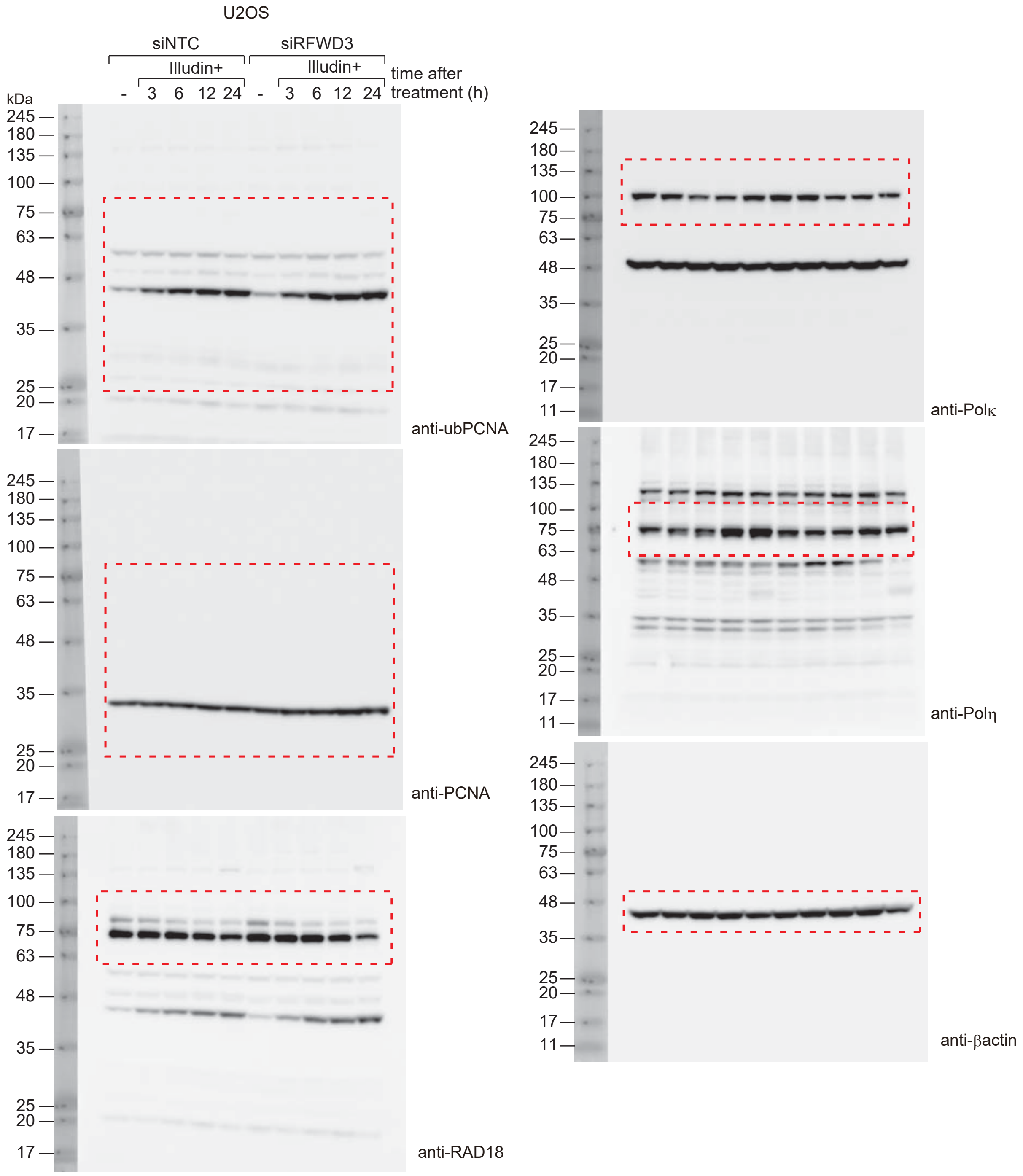

U2OS
siNTC
siRFWD3
Illudin+
Illudin+
- 3 6 12 24 - 3 6 12 24
time after treatment (h)
kDa
245
180
135
100
75
63
48
35
25
20
17
11
anti-ubPCNA
anti-PCNA
anti-RAD18
anti-Polκ
anti-Polη
anti-βactin

Fig. S4E

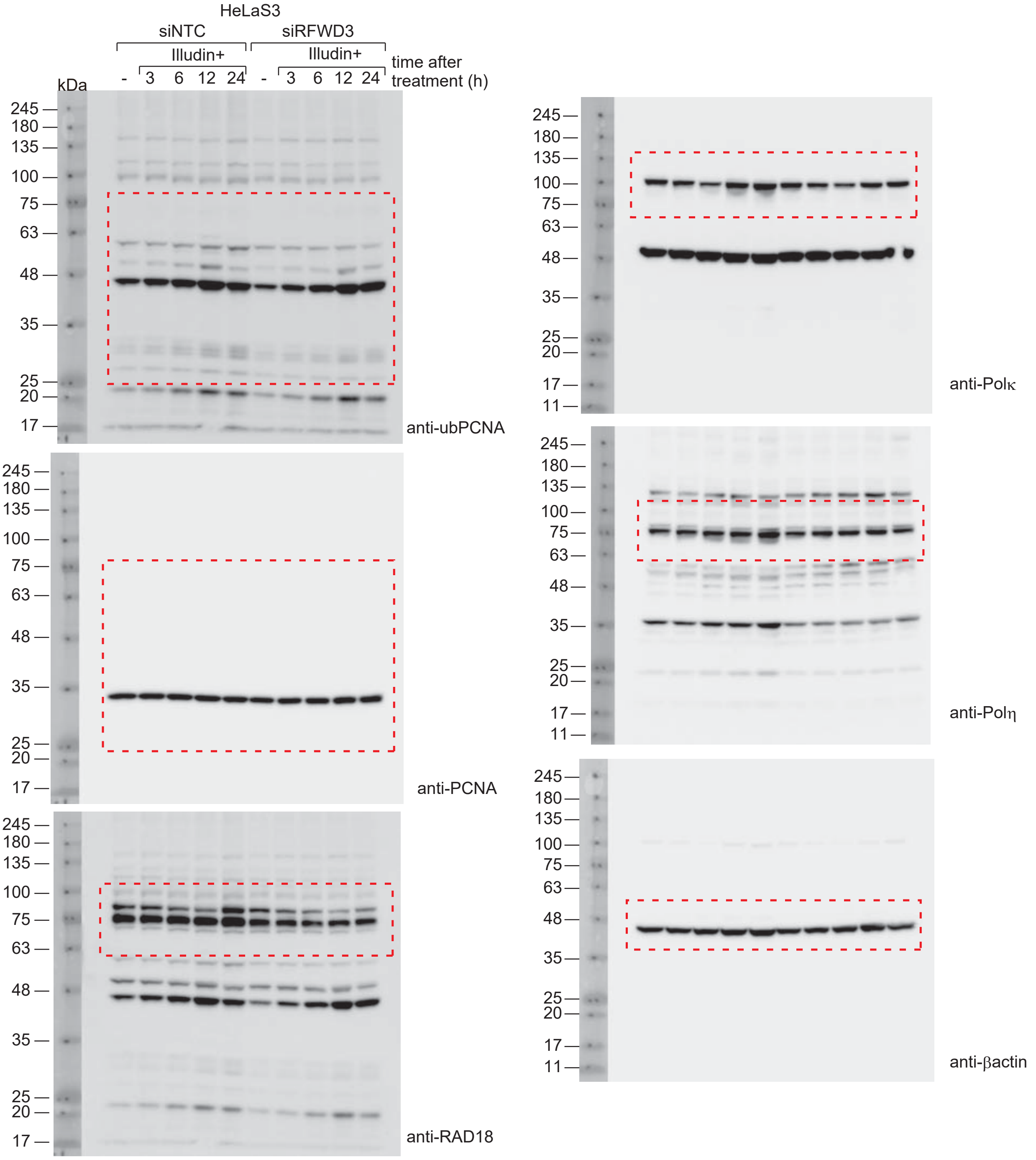
HeLaS3
siNTC
siRFWD3
Illudin+
Illudin+
time after treatment (h)
kDa
- 3 6 12 24 - 3 6 12 24
245
180
135
100
75
63
48
35
25
20
17
11
anti-ubPCNA
anti-PCNA
anti-RAD18
anti-Polκ
anti-Polη
anti-βactin

Fig. S4G

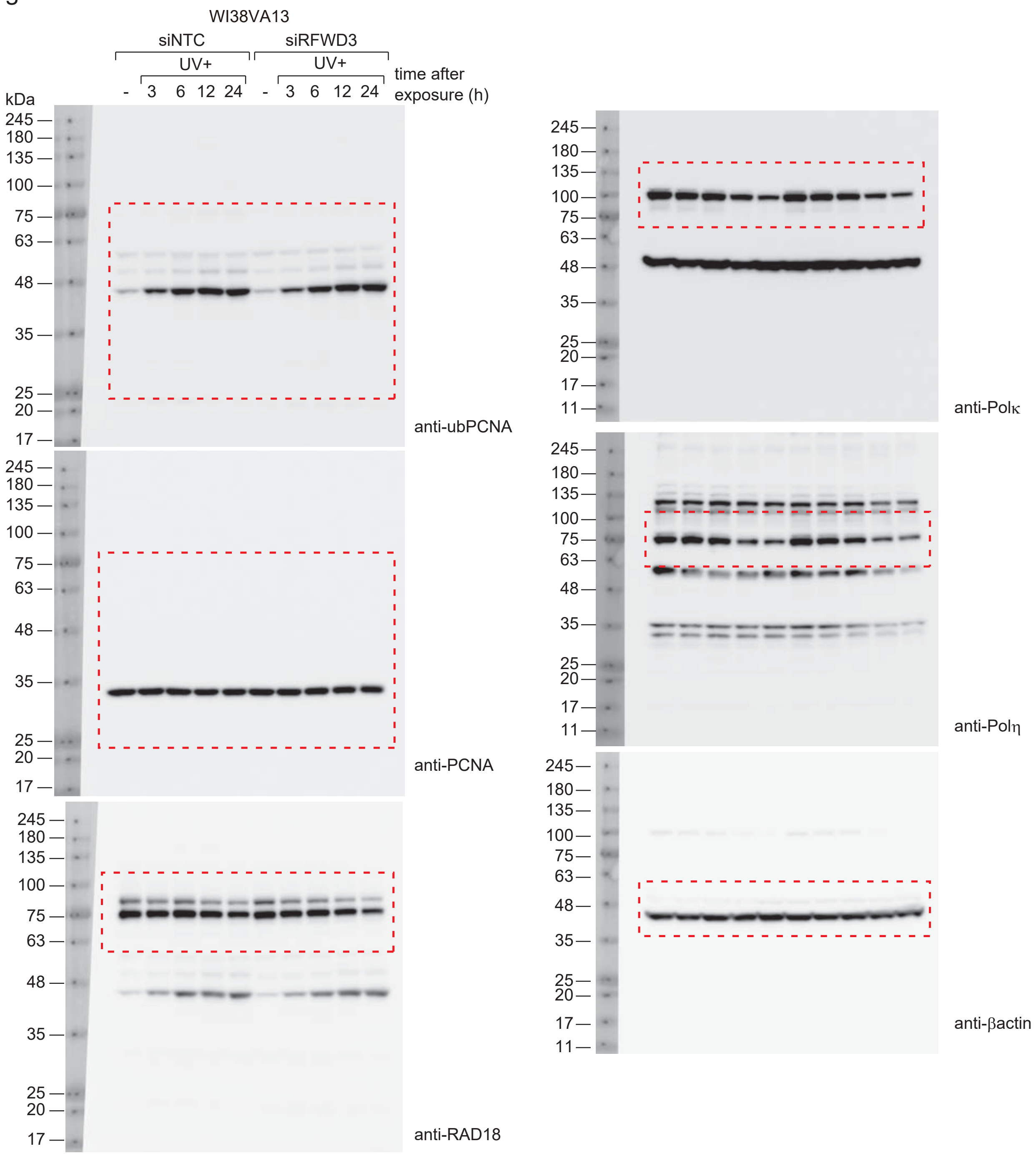

WI38VA13
siNTC
siRFWD3
UV+
UV+
- 3 6 12 24 - 3 6 12 24
time after exposure (h)
kDa
245
180
135
100
75
63
48
35
25
20
17
11
anti-ubPCNA
anti-PCNA
anti-RAD18
anti-Polκ
anti-Polη
anti-βactin

Fig. S4I

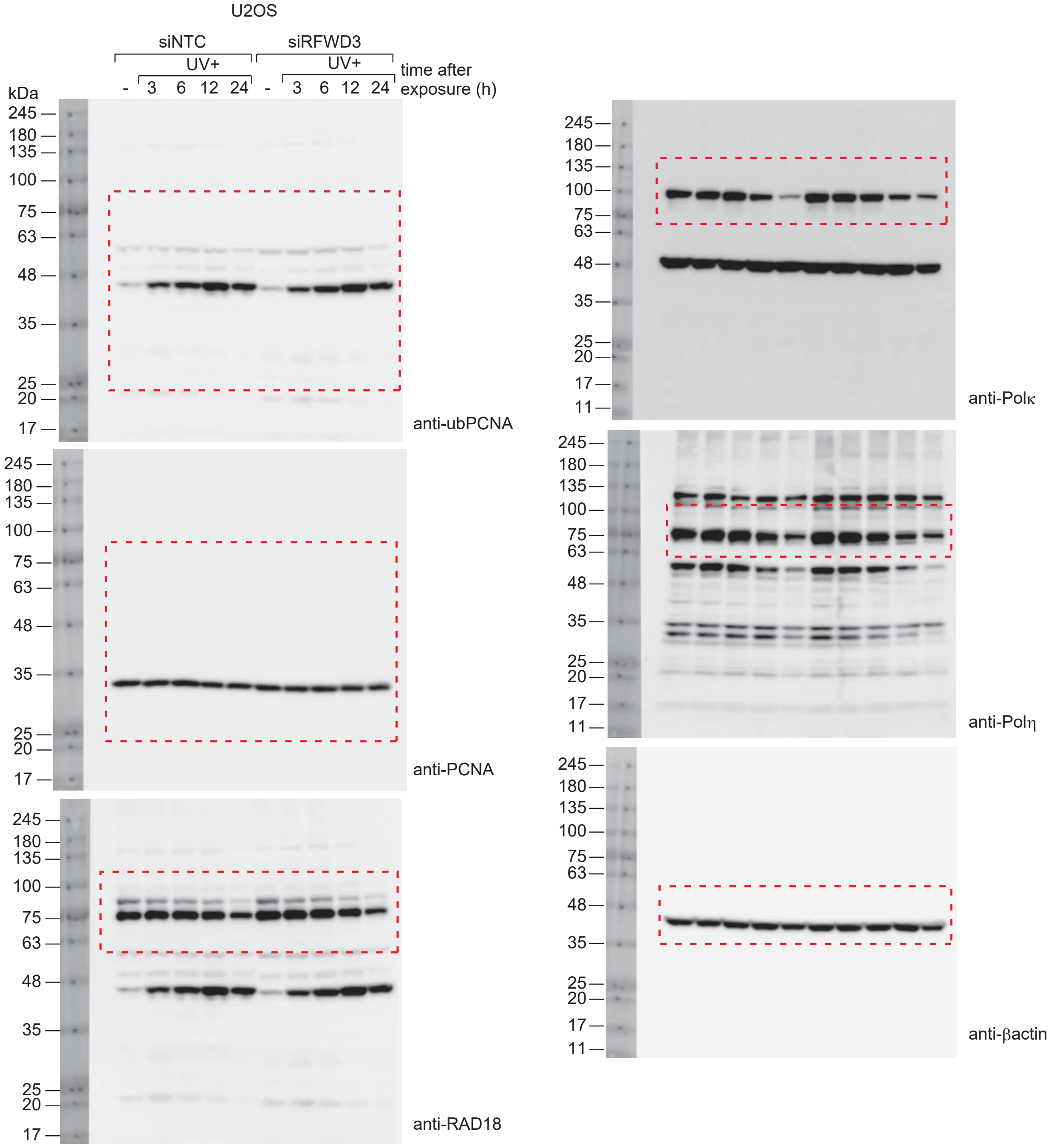
U2OS
siNTC
siRFWD3
UV+
UV+
- 3 6 12 24 - 3 6 12 24
time after exposure (h)
kDa
245
180
135
100
75
63
48
35
25
20
17
11
anti-ubPCNA
anti-PCNA
anti-RAD18
anti-Polκ
anti-Polη
anti-βactin

Fig. S4K

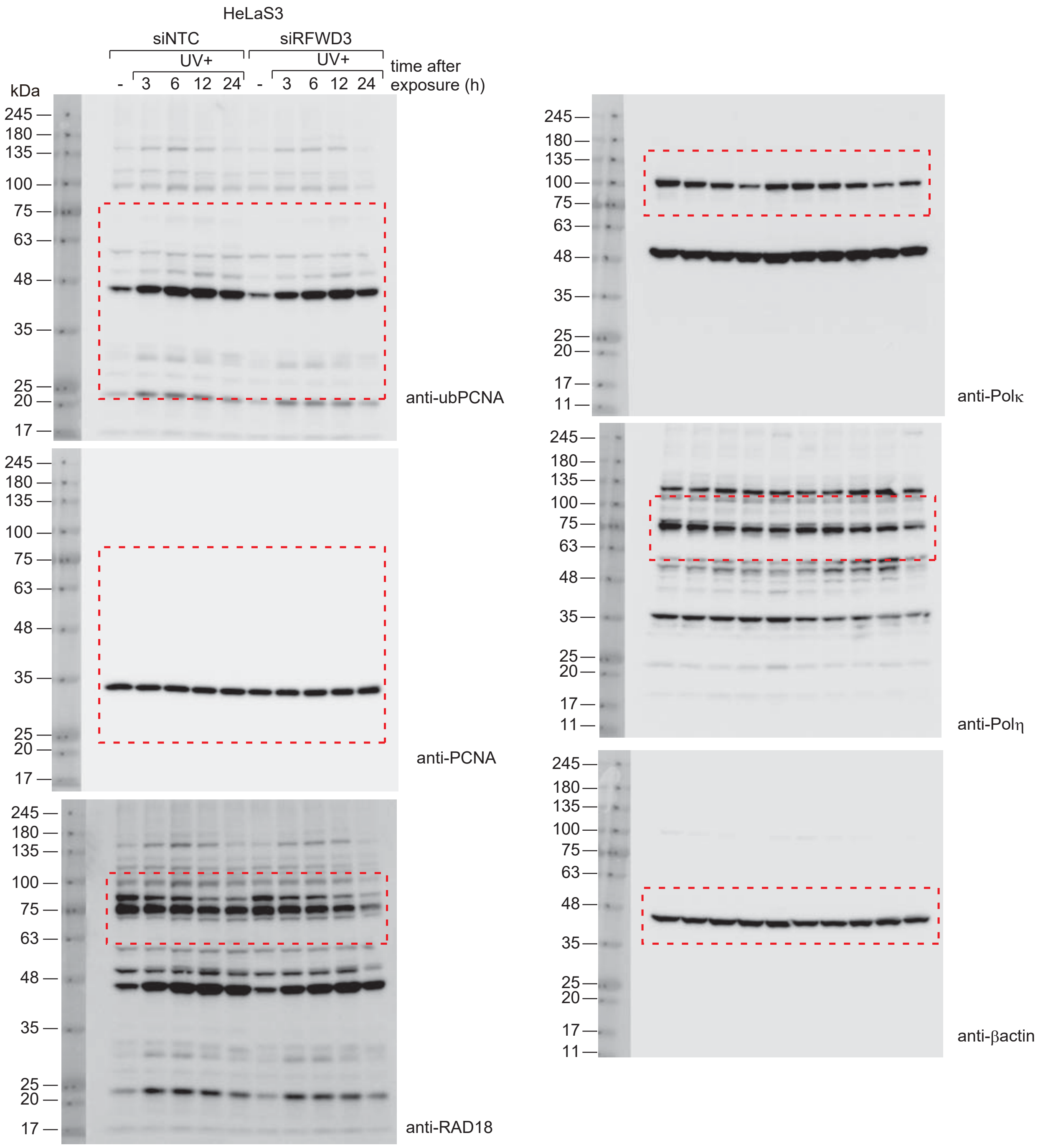
HeLaS3
siNTC
siRFWD3
UV+
UV+
- 3 6 12 24 - 3 6 12 24
time after exposure (h)
kDa
245
180
135
100
75
63
48
35
25
20
17
11
anti-ubPCNA
anti-PCNA
anti-RAD18
anti-Polκ
anti-Polη
anti-βactin

Fig. S5A

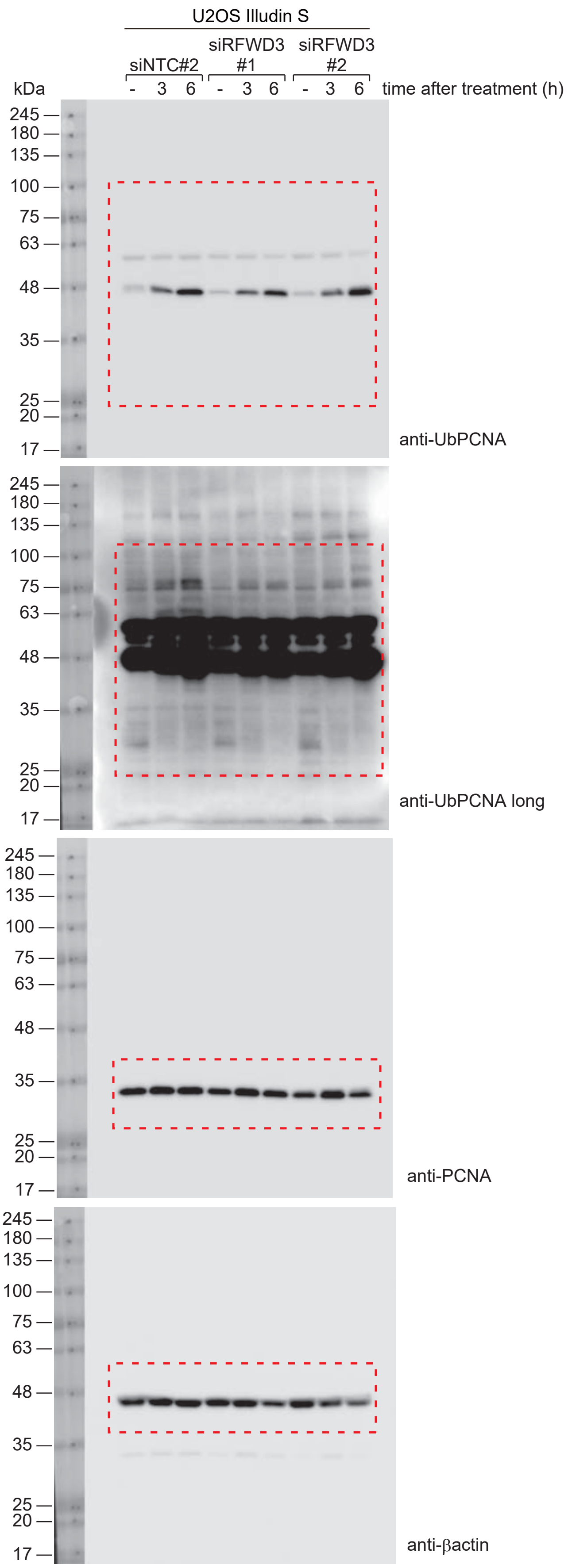

Fig. S5B

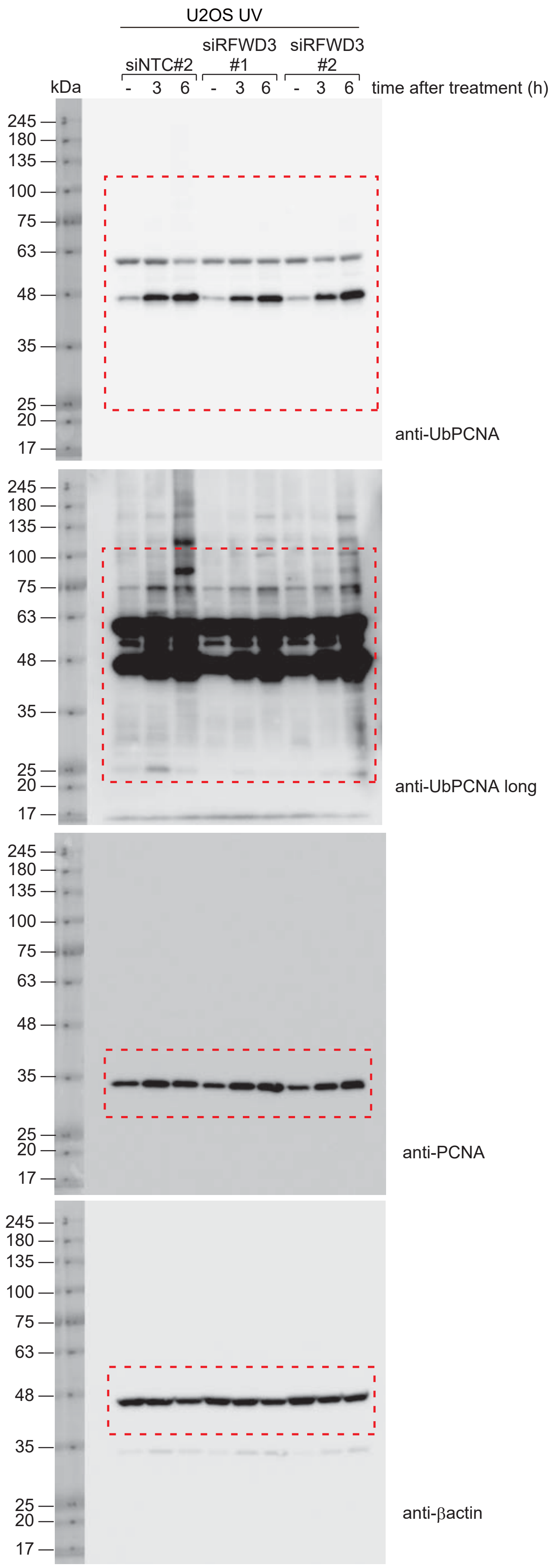

Fig. S5C

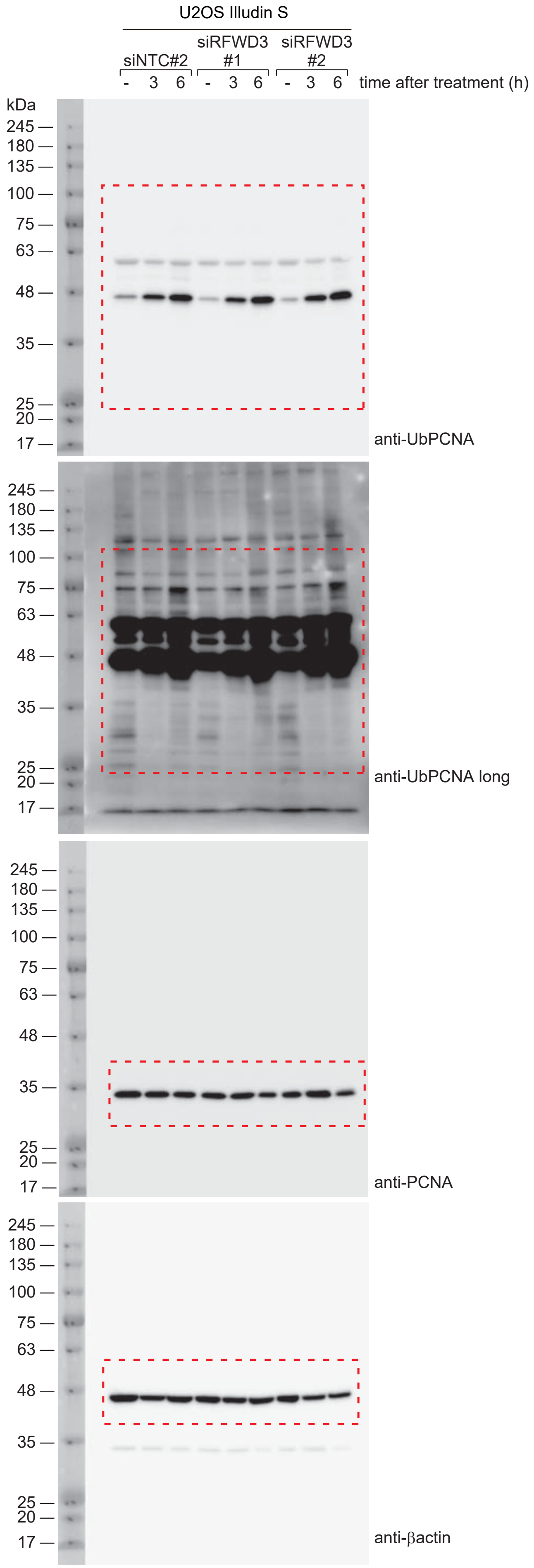

Fig. S5D

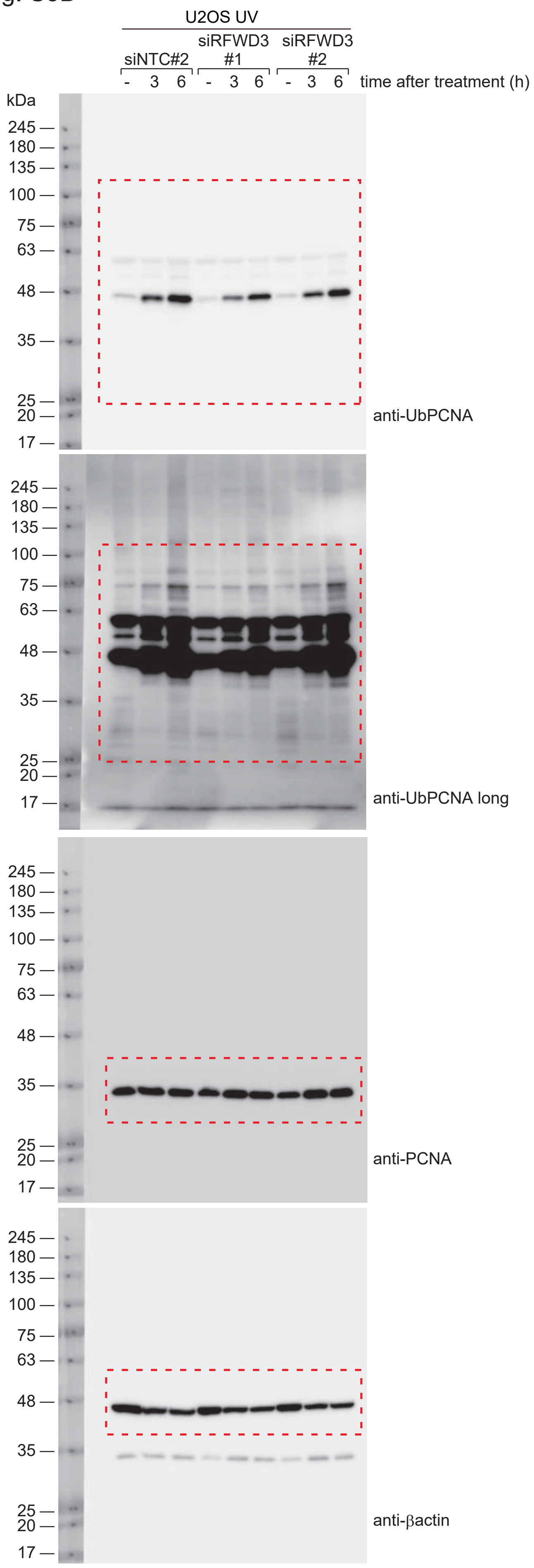

Fig. S5E

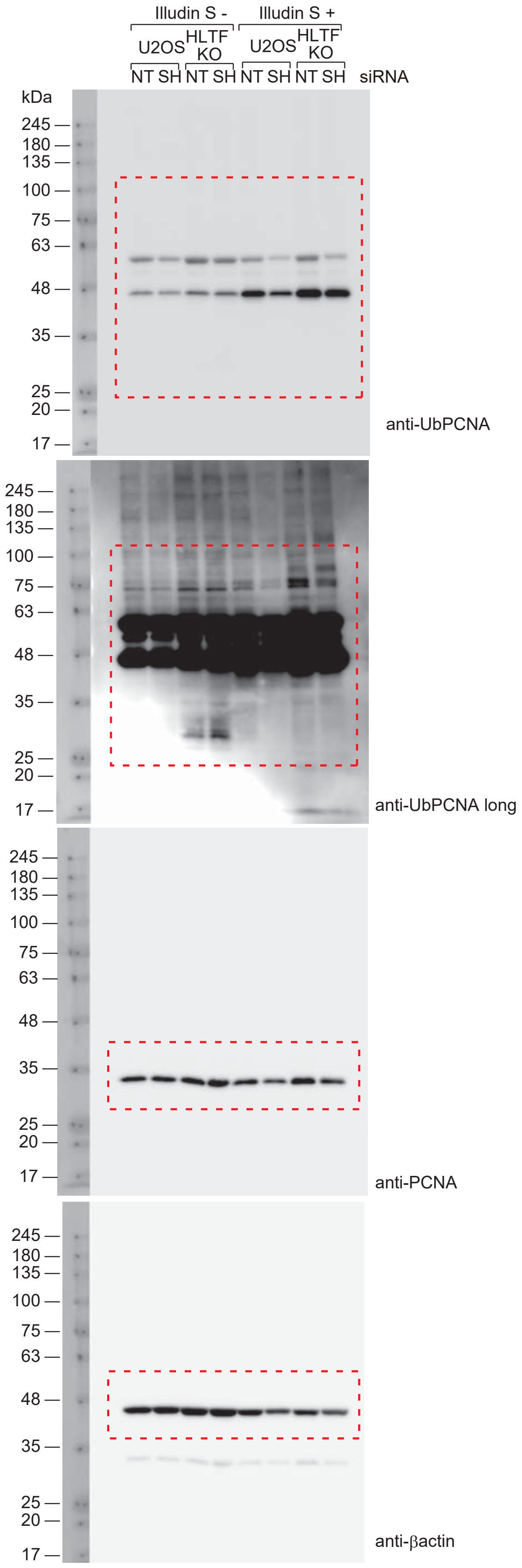
Illudin S -
Illudin S +
U2OS
HLTF KO
U2OS
HLTF KO
NT SH NT SH NT SH NT SH
siRNA
kDa
245
180
135
100
75
63
48
35
25
20
17
anti-UbPCNA
anti-UbPCNA long
anti-PCNA
anti-βactin
