# Supplementary figures and images for: RFWD3 and translesion DNA polymerases contribute to PCNA modification–dependent DNA damage tolerance
Source: Life Sci Alliance. 2022 Jul 29;5(12):e202201584. doi: 10.26508/lsa.202201584 (PMC9348633; doi:10.26508/lsa.202201584)

Fig. 1C

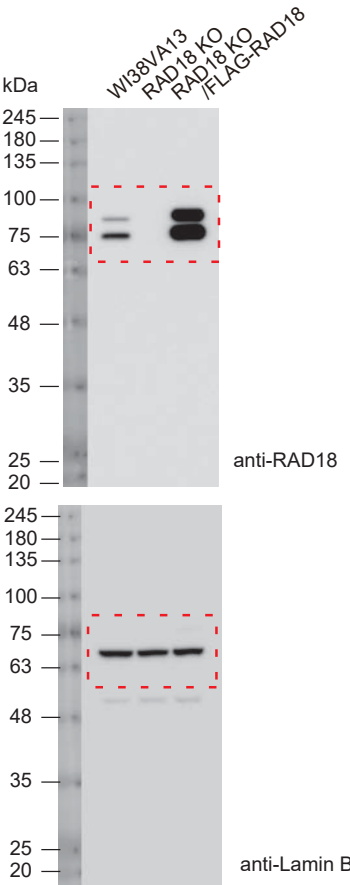

Fig. 1D

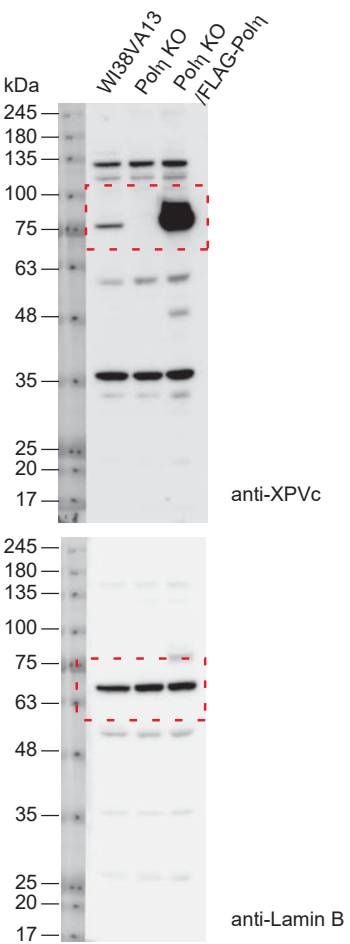

Fig. 1F

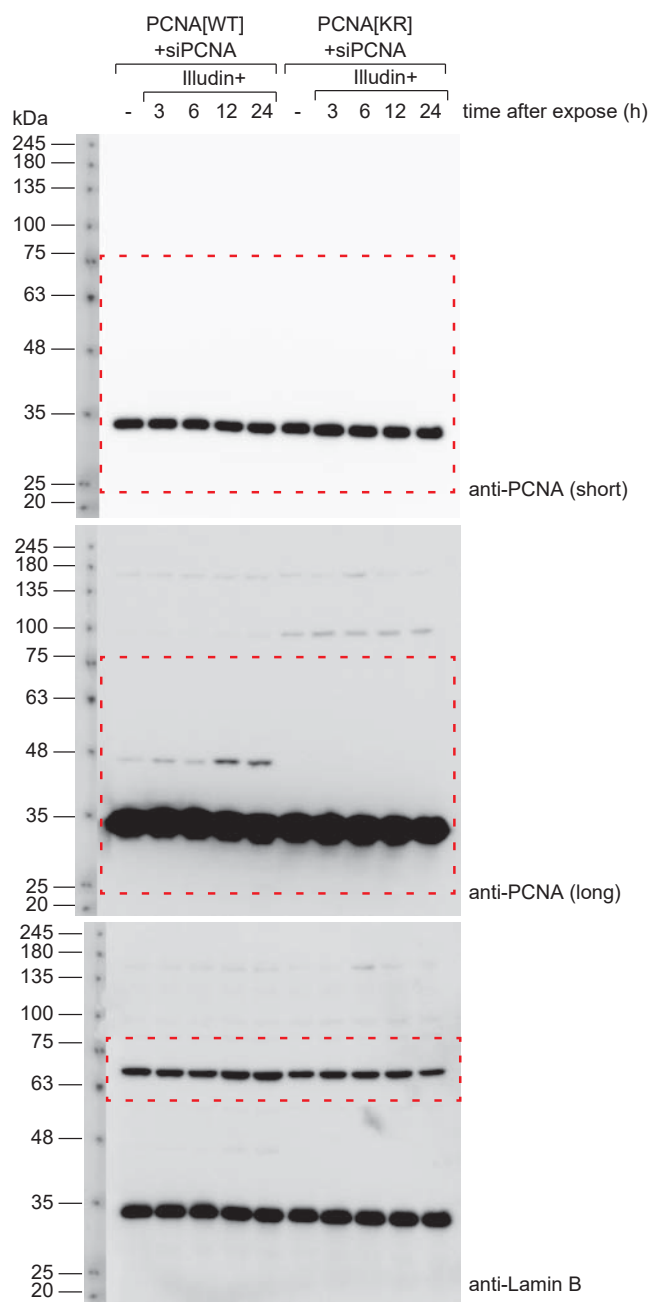

Fig. 1G

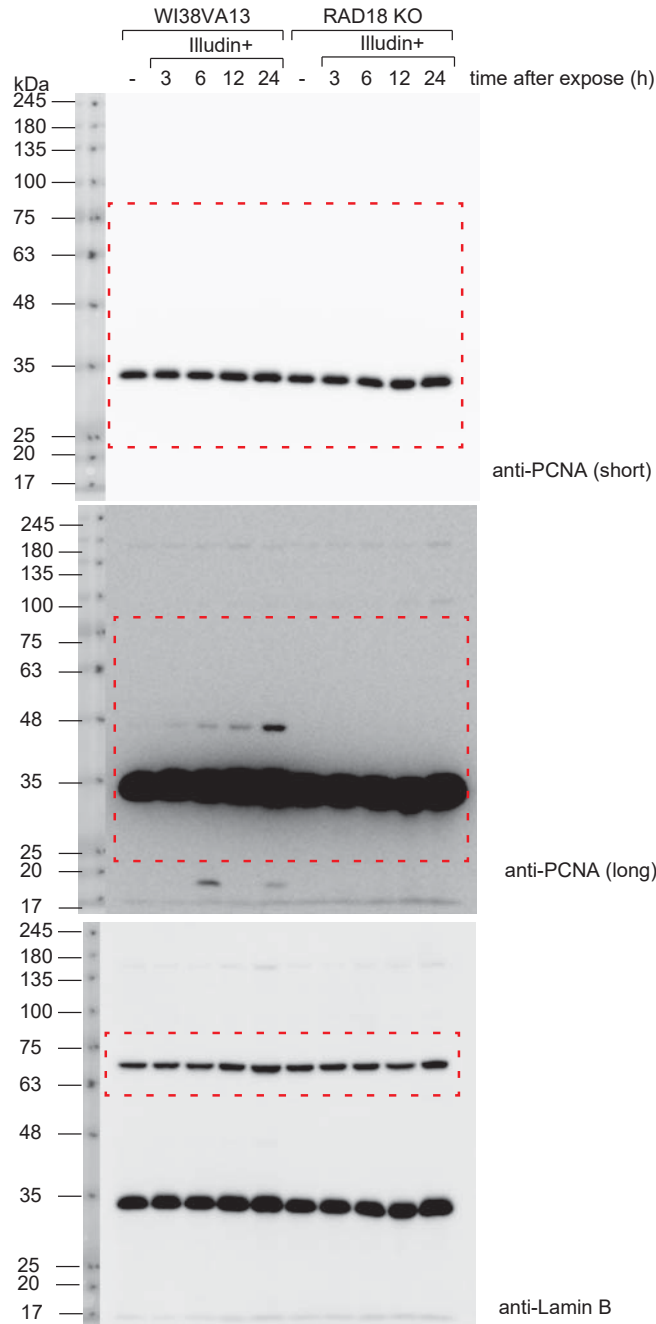

Fig. 21

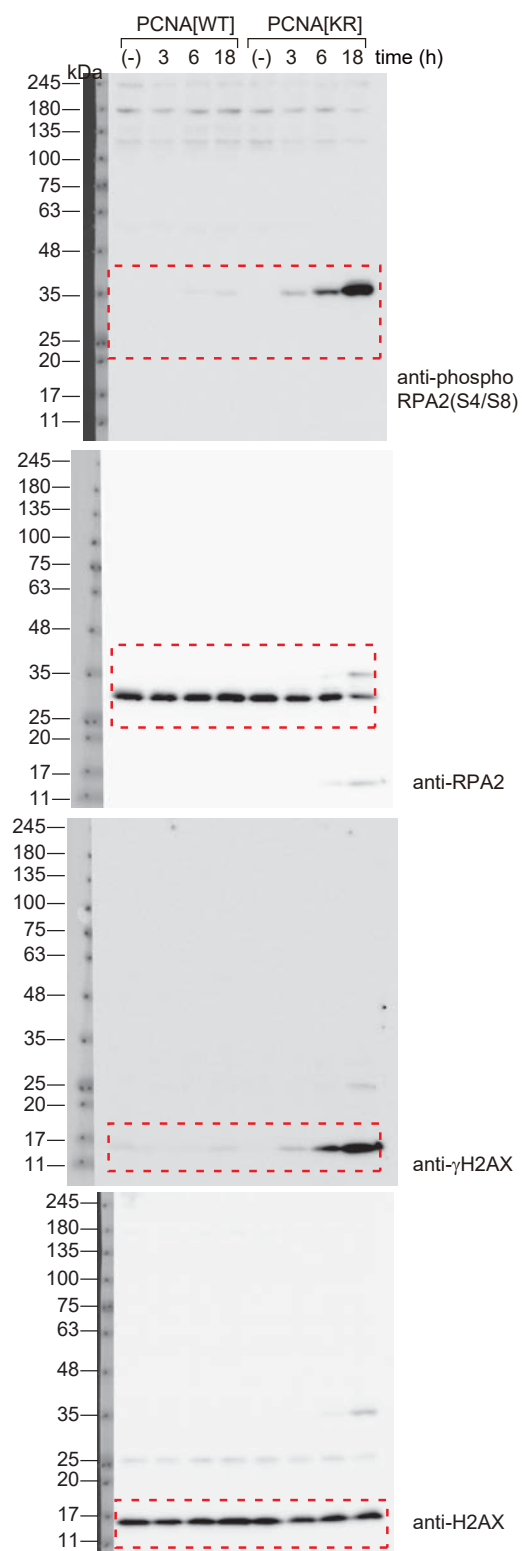

Fig. 3B

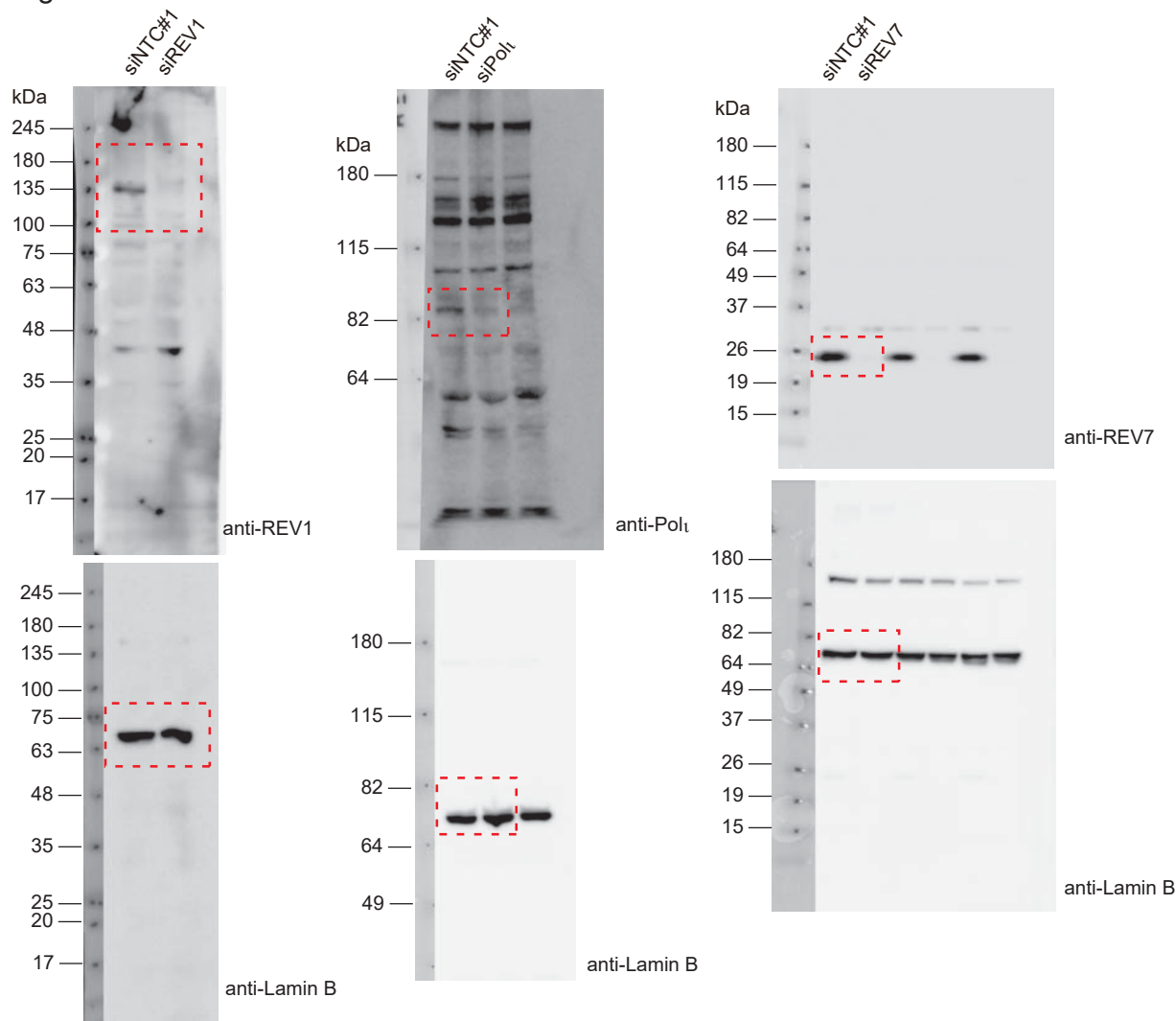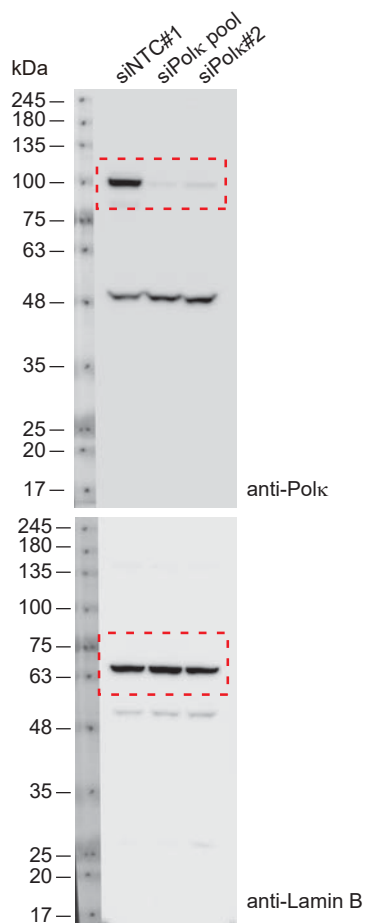

Fig. 3D

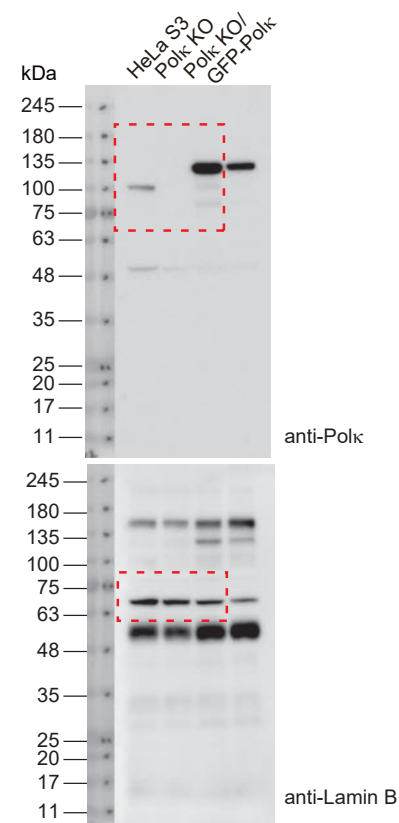

Fig. 3O

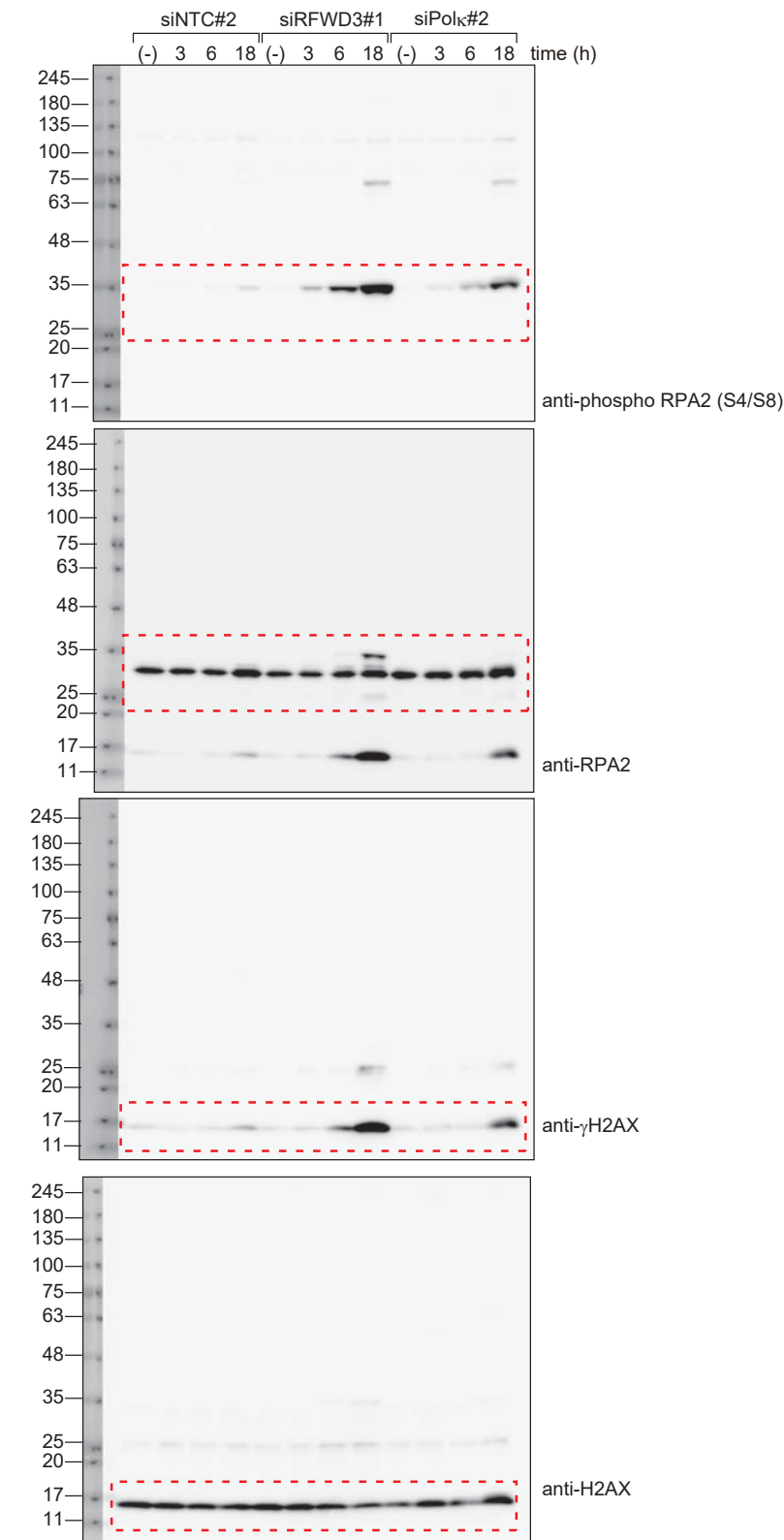

Fig. 3F

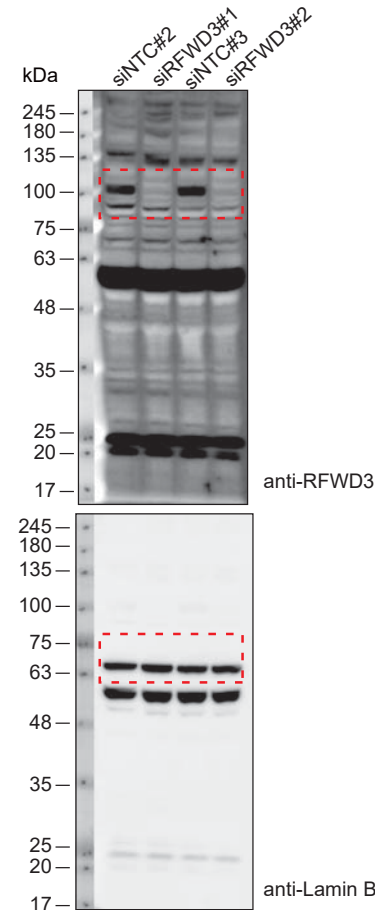

Fig. 4B

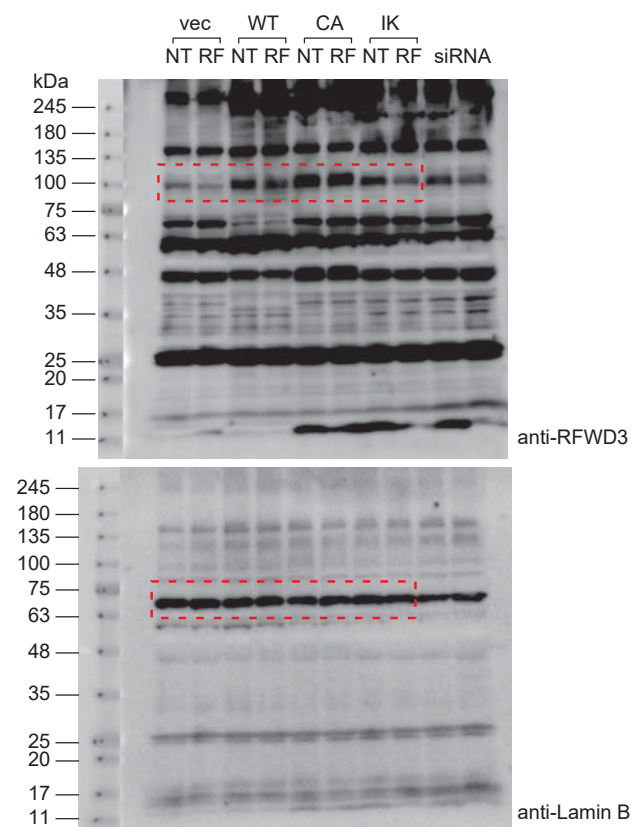

Fig. 4D

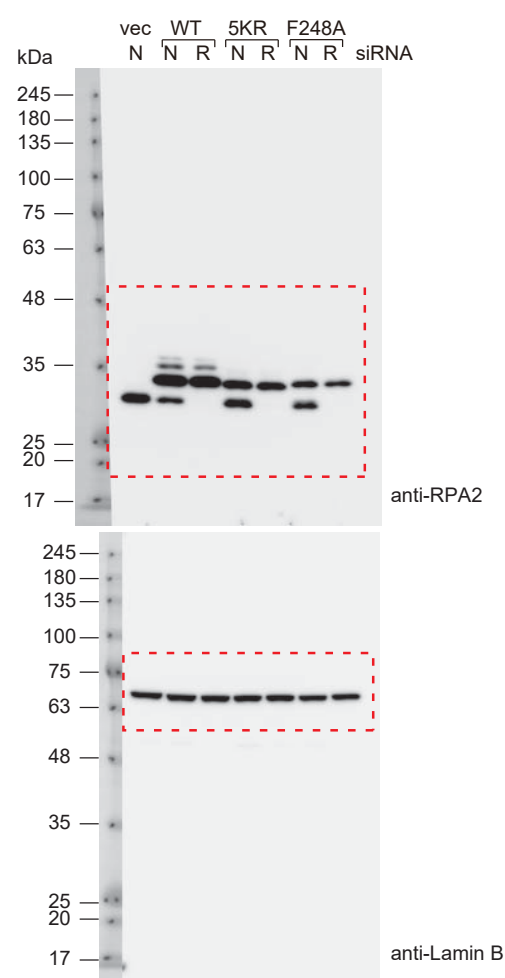

Fig. 5B

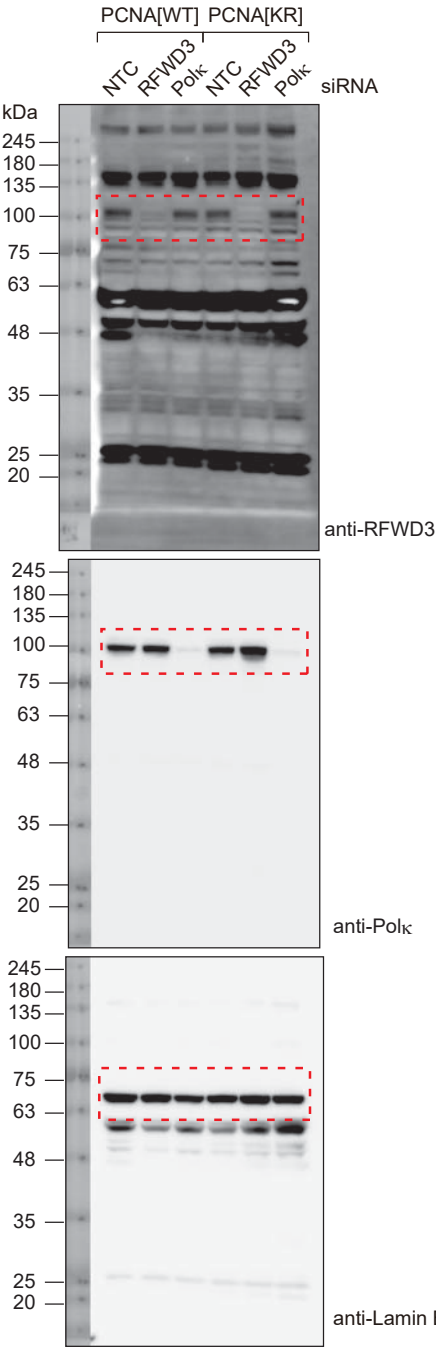

Supplement: Supplementary file 2 [file LSA-2022-01584_SdataF1_F2_F3_F4_F5.pdf]
